# Allostatic Stress and Inflammatory Biomarkers in Transgender and Gender Expansive Youth: Protocol for a Pilot Cohort Study

**DOI:** 10.2196/24100

**Published:** 2021-05-19

**Authors:** Mara Cohen, Baer Karrington, Howard Trachtman, Caroline Salas-Humara

**Affiliations:** 1 Department of Pediatrics New York University Langone New York, NY United States

**Keywords:** transgender, gender diverse, adolescence, allostatic load, stress biomarkers, participatory action research, stress, biomarkers, participatory, gender

## Abstract

**Background:**

A growing number of adolescents are coming out as transgender and gender expansive (TGE). These teenagers have been shown to have significantly worse health outcomes than their cisgender peers. Hypotheses to explain this discrepancy are based on increased stress levels surrounding the societal acceptance of gender identity. In this context, elevated allostatic load (AL), which describes the wear and tear sustained by the body in response to repeated exposure to stress, has been associated with adverse long-term health outcomes.

**Objective:**

This protocol aims to measure AL among TGE adolescents compared with their cisgender peers and assess how AL varies depending on psychological stress and perceived societal acceptance.

**Methods:**

This is an observational proof-of-concept pilot study in which AL will be measured by assaying an array of inflammatory cytokines and cortisol in urine, saliva, and hair samples of TGE youth, and these parameters will be compared with those of age-matched control participants. A questionnaire will assess 4 aspects of psychosocial well-being: presence and management of depression and anxiety, gender identity support by family members, gender minority stress, and degree of perceived safety in the surrounding community. Samples and surveys will be collected at 3 visits (baseline, 6 months, and 12 months). This study will incorporate TGE coinvestigators to inform all aspects of design, data collection, and analysis and ensure that practices are carried out in a respectful and sensitive manner.

**Results:**

As of May 2021, the start of data collection for this project has continued to be postponed as a result of the COVID-19 pandemic, which has both impacted the functioning of the clinic and funding requests. We hope to begin participant recruitment and interviews with coinvestigators soon.

**Conclusions:**

We hypothesize that AL will be primarily influenced by psychological well-being and perceived support and that it will be similar in TGE adolescents and in age-matched cisgender control participants when acceptance and perceived support are high. The results of this study have the potential to increase our understanding of the health challenges faced by TGE individuals during adolescence as well as to show that low levels of acceptance may have detrimental health outcomes secondary to elevated ALs; this may lead to the development of a biomarker profile to assess allostatic stress in TGE patients that can be used to guide management.

**International Registered Report Identifier (IRRID):**

PRR1-10.2196/24100

## Introduction

### Health Disparities in Transgender Youth

On the basis of recent research, it is estimated that between 0.7% and 1.8% of children and adolescents identify as transgender and gender expansive (TGE), the highest percentage of any age group in the United States [1,2]. A TGE individual is a person who is assigned a sex at birth that does not match their current gender identity. Sex refers to someone’s genetic and hormonal makeup (male, female, or intersex), whereas gender is the societally informed expression of assigned sex, and gender identity is someone’s internal sense of gender [3]. Research has shown that TGE adolescents tend to have significantly worse perceived health, higher rates of depression and suicidal ideation, higher rates of HIV, higher rates of experienced violence (childhood sexual abuse and trauma), and fewer preventive health checkups than their cisgender peers [4-9]. Many hypotheses have been proposed to account for these adverse health outcomes, including an increased incidence of bullying and other forms of social rejection [10-13]. The minority stress model is most relevant to this proposal (Meyer [14]), which explains health disparities among sexual minorities and proposes that chronic stress arises from the marginalization, discrimination, rejection, and violence that may be encountered, feared, or internalized. This leads to mental health issues such as depression, anxiety, suicidal ideation and/or attempts, and/or substance abuse [14]. The minority stress model was recently extended to encompass transgender people by including gender-related stressors and transphobia [15,16].

### Allostatic Load

Allostasis is the process to remain stable during change. However, in contrast to homeostasis, in which the internal environment remains steady, allostasis refers to the body’s active fluctuation in response to stressful changing conditions [17]. In response to this stress, the hypothalamic-pituitary-adrenal (HPA) axis and the sympathetic-adrenomedullary axis are both activated and in turn incite many physiologic changes to allow the body to adapt to stress, with primary mediators including neuroendocrine chemical messengers such as cortisol, epinephrine, and norepinephrine, and immune and inflammatory chemical messengers such as interleukin-6 (IL-6) and tumor necrosis factor-α (TNF-α) [18]. These primary mediators in turn exact a secondary physiologic response ranging from metabolic (dyslipidemia, elevated glucose levels, and waist-hip ratio) to cardiovascular (increased blood pressure and heart rate variability) and inflammatory (C-reactive protein [CRP] and fibrinogen) [18,19]. In addition, nutrition and medication use can influence primary and secondary responses [20]. The allostatic load (AL) model is the end product, the cumulative toll of this stress to a certain point in time on physical and mental health to create a comprehensive picture of the body’s response to stress and trauma. This model has been used successfully in children, with recommendations on measuring both primary and secondary end points to best capture both axes [17,21]. IL-6 and CRP were the most frequently measured inflammatory biomarkers based on a systematic review published in 2020 [22]. An elevated AL in the absence of a current stressor represents the *wear and tear* put on the body’s ability to adapt due to repeated exposure to stressful events, whether these events pose a real or interpreted threat [23]. These real or perceived threats trigger the activation of both the sympathetic nervous system adrenal medullary axis and the HPA axis, releasing catecholamines and glucocorticoids, respectively [24]. Although beneficial in the short term, chronic overactivation of these axes can have a detrimental effect on biological systems, leading to overcompensation and eventual collapse as well as decreased ability to respond to future stressors due to changes in the nervous system [17]. One’s resilience to stress and ability to perceive threats and properly mobilize allostatic mechanisms is determined by a combination of many factors, including individual (eg, genetics), behavioral (eg, coping abilities), and historical differences (eg, prior episodes of trauma, abuse, or stressful environments) [17].

Elevated cortisol, in addition to other inflammatory cytokines such as interleukins and TNF-α, is associated with deleterious health consequences [25], such as cardiovascular compromise [26], increased susceptibility to asthma exacerbations [27], and impaired wound healing [28]. An increase in overall AL has also been associated with negative health outcomes later in life. The large-scale American National Health and Nutrition Examination Survey found that AL can serve as a predictor of ischemic heart disease in conjunction with income gradient and education [29]. Seeman et al [30] documented that among older adults, higher baseline AL was associated with increased 7-year mortality and decreased physical and cognitive function. They also found that AL was a better predictor of mortality and physical ability than either looking at specific syndromes or individual stress markers [30]. Furthermore, there is evidence that the effects of increased AL may be particularly significant during key developmental periods, such as adolescence [18].

### AL and the Lesbian, Gay, Bisexual, and Transgender Community

Chronic stress affects the physical well-being of lesbian, gay, and bisexual (LGB) adults, which has been demonstrated in studies showing different baseline cortisol levels and altered cortisol reactivity to stress in LGB adults compared with heterosexual adults [31,32]. However, very few studies have examined the stress biomarkers of TGE people [33]. A study by Colizzi et al [34] of 70 adult transmen and transwomen who had cortisol levels measured before and 12 months after initiating gender-affirming hormones found that the participants had lower cortisol levels after gender-affirming hormone therapy [34]. In addition, DuBois et al [35] explored cortisol levels in transitioning adult transgender men; transmen who reported higher levels of transition-related stressors had higher morning cortisol levels than men who did not report feeling transition-related stressors.

Despite the dearth of studies on stress biomarkers, multiple studies have demonstrated that psychological distress is not inevitable for TGE persons but rather mediated through the degree of social and community acceptance and, in particular, parental affirmation of TGE identity [36]. When TGE children were raised in supportive environments, levels of depression, as determined by parent-filled surveys, did not differ from age-matched cisgender control participants [34,35]. Furthermore, having parents who were interested in a gender-affirming program was a protective factor against depression in TGE youth [37,38]. The positive effects of parental support appear to persist through adulthood based on a study of LGB adults who had improved responses to acute stress compared with LGB adults with less parental support during childhood [39]. Other protective factors include pride in one’s gender identity and strong connections to TGE communities [36].

In addition to the support children receive in their homes, multiple other areas could lead to elevated stress around gender identity, such as the safety of a neighborhood and possible comorbid depression and anxiety [40]. Furthermore, these extrafamilial environments, which also include school, religious, and work environments, had an immense influence over intrafamilial experiences [41], demonstrating the interconnectedness of all environments on the experience of TGE youth. These results highlight the importance of affirmative care in all situations, a stance echoed in a recent policy statement issued by the American Academy of Pediatrics [42].

### Filling in the Gaps

To further explore the importance of affirmative care and social acceptance in TGE youth, we propose using AL measurements in addition to psychosocial surveys to monitor physiological stress. The addition of an array of inflammatory biomarkers is important because it not only provides greater insight into overall stress levels but also underscores the physical consequences of an unsupportive environment that may arise from familial disapproval, unsafe geosocial environments, societal stress or marginalization, and/or comorbid psychiatric disorders. These include anxiety and depression, which most likely arise secondary to gender-related discrimination. To the best of our knowledge, no previous studies have explored the interaction of these factors in TGE youth.

## Methods

### Study Aims

This study aims to (1) determine the AL of TGE youth compared with appropriately matched cis-gender control participants based on assays of inflammatory cytokines and cortisol levels and (2) explore the relationship between supportive environments and communities, supportive families, safe geosocial environments, and well-controlled psychiatric comorbidities on the AL of TGE youth.

### Ethics Approval

This study was approved by the New York University (NYU) Langone Health Center Office of Science and Research Institutional Review Board (IRB).

### Study Participants and Sites

We plan to recruit a total of 80 participants, 40 TGE adolescents and 40 cisgender control participants. TGE participants will include children and adolescents aged between 12 and 18 years who attend the gender clinic at Fink Children’s Ambulatory Care Center at NYU Langone for evaluation of gender dysphoria. This age range was chosen as it reflects the ages of the patients seen at the gender clinic and ensures that participants are at a similar life stage. Although the clinic does see patients aged up to 24 years, young adults aged 18 years and older have more autonomy over their bodies and lives, which may influence their perceived stressors. As this was a pilot study, the only required baseline characteristic for all participants was that they identified as either TGE for the study population or cisgender for the control population. However, patients with preexisting chronic conditions, including obesity (BMI>95%), chronic kidney disease stage 3 or higher, liver disease, chronic infections such as hepatitis B, hepatitis C, HIV, tuberculosis, or autoimmune disorders such as systemic lupus erythematosus or primary immunodeficiency, receiving treatment with steroids, or current smokers will be excluded from the study because these conditions and medications or habits may alter levels of stress and inflammatory biomarkers [43-45]. We will also focus recruitment efforts on patients who have not initiated hormone therapy, although this will not be a requirement. In addition, nongender affirming care medication use will not influence participant selection.

A total of 40 cisgender control participants will be recruited from the adolescent clinic where adolescents are seen for a variety of issues, including adolescent consultative care such as gynecological concerns and sexual health. Patients with chronic diseases or diagnosed infections will be excluded as outlined above for TGE participants. Recruitment of control participants in this subspecialty clinic will minimize biases that might occur when recruiting in other clinics, such as different socioeconomic backgrounds or home locations. Control participants will be matched by age and sex assigned at birth in a 1:1 ratio with TGE participants. These variables were chosen to ensure the correlation between neurologic development and the activity of stress hormone pathways.

TGE participants must be able to return for all 3 visits and fill out all surveys in English, whereas control participants will only be required to attend 1 visit.

### Participant Recruitment

TGE adolescents and control participants will be asked if they are interested in participating in this study before or after their usually scheduled appointment. The full details of the study will be discussed, including details of the questionnaire and the need for hair, urine, and saliva samples. Potential participants will be told that not wanting to partake in the study will not affect current or future medical care. Treating physicians will not be included in the consent process to ensure that patients will be comfortable saying no to study inclusion. As the participants will all be under 18 years of age, they will give assent and their parents and guardians will provide consent. If they wish to withdraw from the study at any point, any sample will be immediately retrieved and destroyed. To maintain the study’s statistical power, we will adopt a recruit to replace the enrollment strategy.

### TGE Coinvestigators

A unique aspect of our study, which will hopefully be commonplace in biomedical research, is the active inclusion of members from the study community, in this case TGE individuals, in the implementation and analysis of the study. There are multiple reasons for this. First, the TGE community has often been misrepresented and mistreated by medicine, especially TGE people from racially minoritized backgrounds; consequently, many TGE people feel uncomfortable and unsafe with health care providers [46]. Including community members on the research team to give insight into respectful research practice will hopefully demonstrate the research team’s willingness to learn and help instill trust between the medical professionals involved and community members. Furthermore, TGE coinvestigators will help identify areas of the study that may be easily misinterpreted or difficult to understand for members of the study population. This insight will be invaluable in ensuring the feasibility of the study and the validity of the findings. Finally, TGE coinvestigators will be able to provide invaluable insight into data interpretation and help guide how the findings can be best implemented to help the community.

TGE coinvestigators, who have not yet been recruited, will include 1 transfeminine and 1 transmasculine person between the ages of 18 and 24 years, which should help prevent any overlap with the study population. The sole requirement is the ability to read and write in English and physically come to the clinic in a COVID-19–safe manner. We will try to involve individuals who have an interest in research and/or medicine and have had limited opportunities to become immersed in the field, therefore recruiting from multiple groups. We will recruit patients at the gender clinic seen by an alternative physician not involved in this study. A community institute that provides gender-affirming services, including mental health and support groups, was also chosen as a recruitment site because of the connection the study team has with the institute and to avoid overlap between the study population and coinvestigators. Finally, we will also recruit from a local shelter for TGE youth who are unhoused. Recruiting from these places will ensure that we are providing all TGE youth who may want to participate in the opportunity to apply. After filling out an initial interest form on Research Enterprise Data Capture (REDCap), accessible with a quick response (QR) code and link on the pamphlet, applicants will be contacted for a phone interview and asked about their availability, goals, and interest in the project. Prior experience with research will not be a requirement because we are particularly seeking individuals with limited previous opportunities. We acknowledge that using a web-based survey for application will limit the opportunity to those who have the technology. However, we believe this will have a minimal effect because most young adults have telephones with the capacity to use QR codes [47].

Although we acknowledge that the TGE coinvestigators were not involved in the research question formation or the original design, and therefore this project cannot be labeled participatory action research, they will be involved in the setup and implementation of the project going forward. TGE coinvestigators will have the opportunity to review the study design and surveys to ensure that they agree that the design will be effective and that there are no overlooked assumptions, leading or poorly worded questions, or other issues with the protocol. If TGE coinvestigators suggest that it may alter previously agreed-upon end points, there will be a research team meeting to discuss the proposal and decide if that end point is needed or discuss an alternative way to reach that end point. During this time, they will undergo the Collaborative Institutional Training Initiative program training, as required by all researchers at the hospital. This training will include Health Insurance Portability and Accountability Act training to help protect research participants’ identities. TGE coinvestigators will be involved in data analysis, helping to provide a community perspective when interpreting the results, and will be involved in writing up and presenting the study results in academic journals and conferences, respectively. The research team has committed to teaching data analysis methods to help TGE coinvestigators with this section, as we feel that imparting specialized knowledge is an important aspect of this project.

We anticipate that being a part of this research team will help these youth personally and professionally in multiple ways, from gaining research experience to mentorship opportunities. TGE people are underrepresented in medicine and research teams, so including them as coinvestigators will help close this gap [48]. Although TGE coinvestigators will receive many different intangible opportunities, we will also compensate them monetarily for their time and effort. This demonstrates that the research team values the commitment from the community, which helps decrease important economic disparities in minority communities and acts as an equalizer to show commitment to inclusion [49]. In addition to monetary compensation, we place a strong emphasis on mentorship. All of the study investigators will make time for TGE coinvestigators to review the project in smaller groups and discuss other aspects of medicine and research in general, as TGE coinvestigators would like.

This novel aspect of the study design has several limitations. Although we would prefer to include more than 2 coinvestigators to obtain a broader range of perspectives, there are limited resources to adequately compensate more individuals who might want to take on this role. As the clinic at which the study will take place is not insular but rather part of a larger institution, steps will be taken to limit exposure to possible institutional transphobia that TGE coinvestigators may face in the workplace. These will include informing staff and security about TGE coinvestigators and their role in the project ahead of time and providing support during onboarding procedures, which may have limited gender options.

### Study Design or Data Collection and Measures

**Figure 1:**
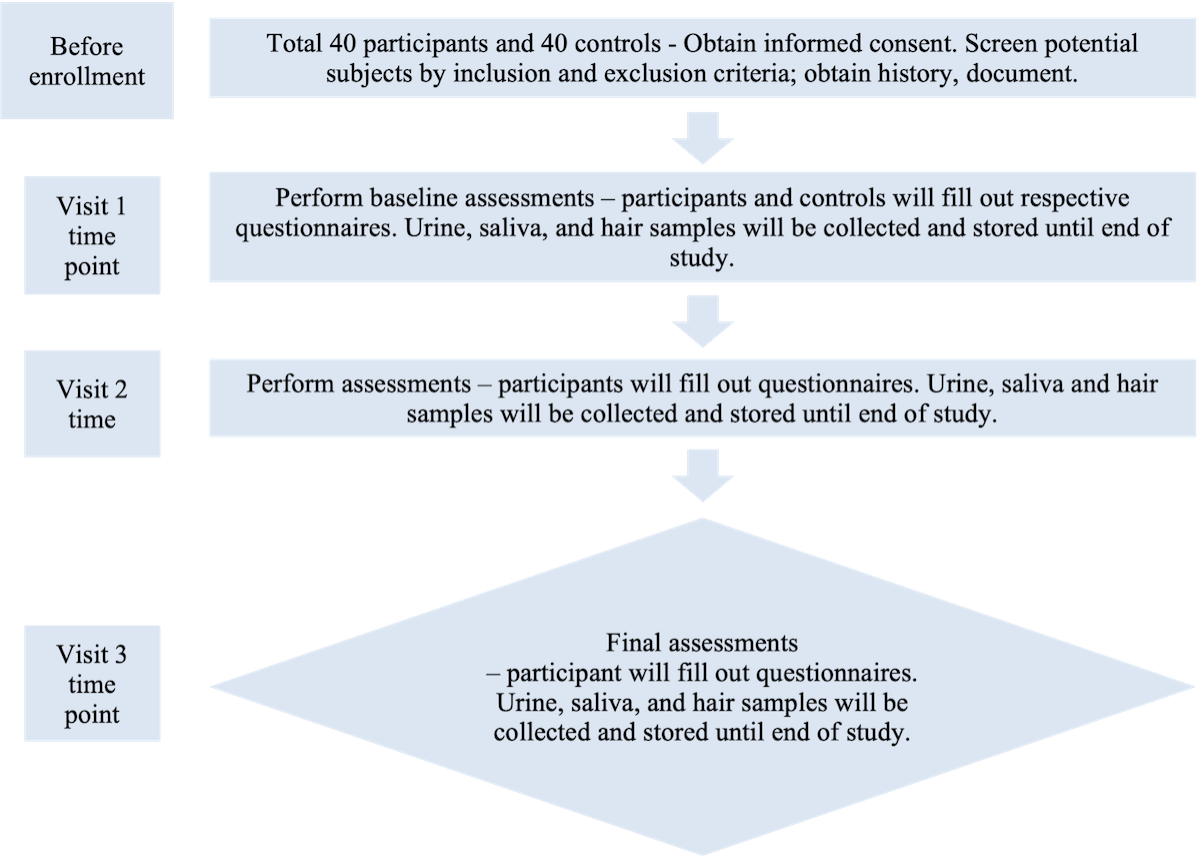
Schematic of the study design. This schematic demonstrates the tasks completed at enrollment and at each subsequent visit for transgender and gender expansive TGE participants.

There will be 3 visits in total for the TGE participants, 6 months apart (baseline, 6 months, and 12 months). The total duration of participation will be 1 year. Figure 1 shows a schematic representation of the TGE arm study design. Control participants will only require a single baseline visit for bio-sample collection and will not be required to take any surveys.

During all visits, the child’s gender identity and 4 main variables—familial support, geosocial safety perception, comorbid depression and anxiety, and gender identity minority stress—will be determined via a survey questionnaire administered on REDCap, which will also store participant data. Participants will be given the Parental Attitudes of Gender Expansiveness Scale for Youth and the Adverse Childhood Experience Questionnaire to determine familial support [50,51]. To determine geosocial safety perception, a previously used questionnaire to inquire about a child’s perception of neighborhood safety will be given [52,53]. Participants will be given the modified Patient Health Questionnaire-9A (adolescent) and Self-Report for Childhood Anxiety Related Emotional Disorders survey [54] to assess depression and anxiety. Finally, gender-related discrimination, rejection, and victimization experienced internally and externally will be surveyed with the gender minority stress and resilience (GMSR) measure modified to be suitable for adolescents [55]. The GMSR also addresses intersectionality and how gender identity may be perceived with other identities, such as religious, race or ethnicity, or occupational identities, as well as addresses community support by exploring how welcomed individuals feel by their communities. All surveys used have strong reliability and validity, and each participant has approximately 150 questions to complete. These surveys are expected to take approximately 1 hour to complete. A study investigator will be nearby to help participants in the survey if needed. All surveys used except for the GMSR have been validated in children aged 12 years and older. Although the GMSR has not been validated in people younger than 18 years old, it is the only measure of gender minority stress of which the authors are aware. The question responses were modified to be appropriate for participants aged between 12 years and 18 years. The research team recognizes that answering 150 questions can be demanding, especially for young people, and the risk of decreased attentiveness as the questions continue can affect the results. To help prevent this, snacks and beverages will be provided. In addition, every approximately 20 minutes until completion, participants will be asked to stand up, walk, and/or stretch. Participants will have the option of asking for the accompaniment of a research team member should they desire. Insurance information will be collected from the electronic health record, which will indicate the family’s access to medical care.

Stress and inflammatory biomarkers will be measured at the initial visit and the 2 follow-up visits. To capture the primary mediators of AL, the biomarkers measured were cortisol, IL-6, and TNF-α. The secondary mediators measured included CRP, BMI, and waist-to-hip ratio. Measuring both primary and secondary mediators is recommended in pediatric patients to best capture the AL [21]. Efforts will be made to schedule all appointments during the afternoon to minimize diurnal variation and enable a more uniform assessment of cortisol and urine inflammatory markers levels [43,56-58]. At each session, participants will have stress and inflammatory biomarkers measured before the survey and after listening to a 10-minute relaxation tape to account for possible transient increases in stress on route to the appointment. Researchers will be blinded to the sample source to reduce possible bias.

Urine samples will be collected and assayed using the Luminex Panel (model number HSTCMAG28PMX21BK), which has been used successfully to determine inflammatory and stress biomarkers in infants and toddlers enrolled in an obesity prevention trial [59]. This panel can measure an array of cytokines, including IL-1ß, IL-6, IL-10, and TNF-α. Salivary and hair cortisol levels will also be measured. Salivary cortisol has been used previously in adolescent populations to explore the effects of parental support on stress response [60]. Hair cortisol content is an integrated index of stress over an extended period. A small sample of hair from the head will be collected with child-safe scissors as a measure of chronic stress levels [61,62]. Saliva will be collected into a polypropylene vial with a straw using the *passive-drool technique* [63]. All samples, marked with the participant ID number, will be stored at −70 °C within 1 hour of collection until the batch assay of the analyte panels. If agreed upon by participants, the residual samples on will be stored in the NYU Biorepository for potential use in future studies. Otherwise, all samples will be destroyed after the completion of the study.

### Sample Size

Recruitment will end when approximately 40 TGE participants and 40 control participants are enrolled for a total of 80 participants, as determined by a total of approximately 200 patients in the gender clinic with an expected enrollment of 20% to 30%. On the basis of a total population of approximately 21,000 transgender youth in New York State from the Williams Institute and a total of 200 patients in our clinic, a sample population of 13 participants and 13 control participants would be adequate to generate a study with 80% power with a 95% CI of 17 [34]. Therefore, by aiming for 40 participants in each arm, we will exceed this value.

### COVID-19 Precautions

This pilot study was conceptualized before the COVID-19 pandemic. Most of this study will not be affected. Participants will be able to complete the survey on a computer either by themselves or with a single research team member in the room, which will allow COVID-19 safety precautions. Sample collection will also be done with a single research team member in compliance with COVID-19 precautions. The recruitment of TGE coinvestigators will be done using fliers and pamphlets, as previously discussed, and interviewing TGE coinvestigators will be performed over Zoom, Skype, or FaceTime. Although originally the TGE coinvestigators were to be a part of data collection, because of COVID-19 restrictions, this will not be possible. However, the research team will keep TGE coinvestigators abreast of implementation and be available to discuss data collection.

COVID-19 has added numerous stressors to everyday life, and as such, has most likely caused changes to individual ALs. However, no studies have explored COVID-19 and ALs in pediatric patients to the research team’s knowledge. The control group will correct for any universal increases. If another major shift in the pandemic should occur during the study period, then the control group will have an additional measurement session added to adjust for how this shift may affect ALs.

### Statistical Analysis

#### Primary Objective

Our general approach of comparing TGE youth biomarkers with cisgender control participants will include paired two-tailed *t* test analysis and analysis of variance (ANOVA) to determine whether there is a significant difference in the cytokine profile between the 2 groups’ levels, as defined by a *P* value of less than .05.

#### Secondary Objective

Within the TGE youth samples, we will use paired *t* test analysis and ANOVA to determine if there is a difference in biomarker levels between different age brackets (12-14 years and 15-18 years) and ethnicity (White vs Black or Hispanic), with significance being determined by a *P* value less than .05. If we do not have enough participants in a certain age bracket, we will not perform this analysis.

Linear regression analysis will be conducted to determine if there is a relationship between questionnaires and biomarker levels for each participant. We will also include a regression analysis to ascertain correlation, with an R-squared value greater than 50% needed to determine the relationship.

### Statistical Execution

All statistical calculations will be performed using GraphPad Prism version 8.0.0 for Mac (GraphPad Software).

## Results

As of September 2020, the study received IRB approval but had not yet begun data collection. Although we planned to begin active recruitment in the summer of 2020, we have had to postpone owing to the COVID-19 pandemic. This health emergency has significantly affected the frequency of in-person visits and clinical research. In addition, we are still waiting to hear from multiple grants that were delayed as a result of the pandemic. We hope to receive funding support and begin participant recruitment and interviews with coinvestigators shortly.

## Discussion

### Summary

As described above, adolescents have the highest percentage of TGE identifying members of any age group in the United States, yet this population has minimal published research [1,2]. This study seeks to fill some of the gaps in the literature regarding the physical consequences of acceptance. We propose that AL of TGE individuals may be used as an integrated biomarker for the risk of long-term adverse health consequences based on the level of acceptance in different environments. To reiterate, we hypothesize that TGE status is not a cause of increased AL, but rather that an increase is the consequence of social and environmental experiences. To this end, we therefore sought to measure the presence of 4 variables—depression and anxiety, gender identity familial support, gender identity community support, and gender-related minority stress—which may influence the AL in TGE youth. The effects of psychiatric, familial, and community support on AL may help target areas of effective intervention going forward and provide a better understanding of the stress sources among TGE youth.

### Strengths and Limitations

This study has some limitations that must be acknowledged. The confounding variable of socioeconomic status, which can increase stress, is outside the scope of this study. By recruiting all patients from the same site, there were some limitations to the range of backgrounds represented. However, given the diversity of the area surrounding the hospital and the limited number of sites providing adolescent gender care in the New York metropolitan area, we expect to be able to recruit a diverse range of participants. Furthermore, the fact that participants are already seeking care at an adolescent gender clinic can bias the sample toward more accepting situations. We hope that we can still identify subtle variations in levels of support and validation by using a number of surveys. Although the results of this study are limited by sample size, they will provide the first data set that can hopefully be expanded over time as more research is conducted with the TGE youth population.

Another area of potential concern is the direct effect of gender-affirming therapy on the results. However, we believe that this effect, even longitudinally, should be minimal. There are no consistent data indicating that exogenous hormones influence the HPA axis [34,64-66]. Colizzi et al [34] reported that estrogen might alter hormone-binding globulin levels, thereby skewing stress hormone levels. In addition, brain plasticity, synapse formation, and signaling pathways differ between male and female rodents based on sex hormone levels, with some indication that a similar process could occur in the human brain [64]. However, in a study of 20 postmenopausal cisgender women, exogenous estrogen was associated with only slightly elevated levels of dehydroepiandrosterone and dehydroepiandrosterone sulfate and not significantly elevated levels of cortisol [65]. In a separate study of 12 cisgender women, exogenous testosterone was found not to influence endogenous cortisol levels [66]. Although commencement of gender-affirming therapy at any point during the study should not significantly alter our data, we will document gender-affirming hormone use to enable later adjustment for any effect. Furthermore, the effects of gender-affirming therapy on self-perceived stressors will be captured during our surveys.

### Conclusions

The results of this study will be useful in guiding larger-scale studies to assess allostatic stress in TGE youth. These results also have the potential to shape practical interventions to benefit TGE youth by providing scientific evidence in favor of affirming programs, including support groups for both parents and teenagers, parental consultations with gender experts with a range of backgrounds to increase familial acceptance, encouragement of Gay-Straight Alliance and LGB club involvement, and encouragement of community centers with a TGE focus. In addition, the results of this study have the potential to enhance the argument for policies that would increase TGE safety and acceptance in the community, such as antibullying measures. The implications of physical harm caused by a lack of acceptance will hopefully increase the trend toward broader acceptance of the TGE population. Finally, the findings may enable the development of a biomarker profile that can be used as an objective measure of allostatic stress in TGE patients and to monitor the effectiveness of therapeutic interventions on their well-being.

## Abbreviations

AL: allostatic load
ANOVA: analysis of variance
CRP: C-reactive protein
GMSR: gender minority stress and resilience
HPA: hypothalamic-pituitary-adrenal
IL-6: interleukin-6
IRB: Institutional Review Board
LGB: lesbian, gay, and bisexual
NYU: New York University
QR: quick response
REDCap: Research Enterprise Data Capture
TGE: transgender and gender expansive
TNF-α: tumor necrosis factor α

## References

[1] Herman JL, Flores AR, Brown TN, Wilson BD, Conron KJ (2017). Age of individuals who identify as transgender in the United States. The Williams Institute, Los Angeles, CA.

[2] Johns MM, Lowry R, Andrzejewski J, Barrios LC, Demissie Z, McManus T, Rasberry CN, Robin L, Underwood JM (2019). Transgender identity and experiences of violence victimization, substance use, suicide risk, and sexual risk behaviors among high school students - 19 states and large urban school districts, 2017. MMWR Morb Mortal Wkly Rep.

[3] Fausto-Sterling A (2000). Sexing the body: gender politics and the construction of sexuality. Choice Rev Online.

[4] Rider GN, McMorris BJ, Gower AL, Coleman E, Eisenberg ME (2018). Health and care utilization of transgender and gender nonconforming youth: a population-based study. Pediatrics.

[5] Perez-Brumer A, Day JK, Russell ST, Hatzenbuehler ML (2017). Prevalence and correlates of suicidal ideation among transgender youth in California: findings from a representative, population-based sample of high school students. J Am Acad Child Adolesc Psychiatry.

[6] Almeida J, Johnson RM, Corliss HL, Molnar BE, Azrael D (2009). Emotional distress among LGBT youth: the influence of perceived discrimination based on sexual orientation. J Youth Adolesc.

[7] Garofalo R, Deleon J, Osmer E, Doll M, Harper GW (2006). Overlooked, misunderstood and at-risk: exploring the lives and HIV risk of ethnic minority male-to-female transgender youth. J Adolesc Health.

[8] Clark H, Babu AS, Wiewel EW, Opoku J, Crepaz N (2017). Diagnosed HIV infection in transgender adults and adolescents: results from the National HIV Surveillance System, 2009-2014. AIDS Behav.

[9] Newcomb ME, Hill R, Buehler K, Ryan DT, Whitton SW, Mustanski B (2020). High burden of mental health problems, substance use, violence, and related psychosocial factors in transgender, non-binary, and gender diverse youth and young adults. Arch Sex Behav.

[10] Roberts AL, Rosario M, Slopen N, Calzo JP, Austin SB (2013). Childhood gender nonconformity, bullying victimization, and depressive symptoms across adolescence and early adulthood: an 11-year longitudinal study. J Am Acad Child Adolesc Psychiatry.

[11] Toomey RB, Card NA, Casper DM (2014). Peers' perceptions of gender nonconformity: associations with overt and relational peer victimization and aggression in early adolescence. J Early Adolesc.

[12] Roberts AL, Rosario M, Corliss HL, Koenen KC, Austin SB (2012). Childhood gender nonconformity: a risk indicator for childhood abuse and posttraumatic stress in youth. Pediatrics.

[13] Russell ST, Ryan C, Toomey RB, Diaz RM, Sanchez J (2011). Lesbian, gay, bisexual, and transgender adolescent school victimization: implications for young adult health and adjustment. J Sch Health.

[14] Meyer IH (2003). Prejudice, social stress, and mental health in lesbian, gay, and bisexual populations: conceptual issues and research evidence. Psychol Bull.

[15] Hendricks ML, Testa RJ (2012). A conceptual framework for clinical work with transgender and gender nonconforming clients: an adaptation of the Minority Stress Model. Prof Psychol Res Pr.

[16] Miller LR, Grollman EA (2015). The social costs of gender nonconformity for transgender adults: implications for discrimination and health. Sociol Forum (Randolph N J).

[17] Juster R, McEwen BS, Lupien SJ (2010). Allostatic load biomarkers of chronic stress and impact on health and cognition. Neurosci Biobehav Rev.

[18] Beckie TM (2012). A systematic review of allostatic load, health, and health disparities. Biol Res Nurs.

[19] Guidi J, Lucente M, Sonino N, Fava G (2021). Allostatic load and its impact on health: a systematic review. Psychother Psychosom.

[20] Suvarna B, Suvarna A, Phillips R, Juster R, McDermott B, Sarnyai Z (2020). Health risk behaviours and allostatic load: a systematic review. Neurosci Biobehav Rev.

[21] Condon EM (2018). Chronic stress in children and adolescents: a review of biomarkers for use in pediatric research. Biol Res Nurs.

[22] Kuhlman KR, Horn SR, Chiang JJ, Bower JE (2020). Early life adversity exposure and circulating markers of inflammation in children and adolescents: a systematic review and meta-analysis. Brain Behav Immun.

[23] McEwen B, Stellar E (1993). Stress and the individual. Mechanisms leading to disease. Arch Intern Med.

[24] Sapolsky RM (2000). How do glucocorticoids influence stress responses? Integrating permissive, suppressive, stimulatory, and preparative actions. Endocr Rev.

[25] Björntorp P, Rosmond R (2000). Obesity and cortisol. Nutrition.

[26] Whitworth JA, Williamson PM, Mangos G, Kelly JJ (2005). Cardiovascular consequences of cortisol excess. Vasc Health Risk Manag.

[27] Marin TJ, Chen E, Munch JA, Miller GE (2009). Double-exposure to acute stress and chronic family stress is associated with immune changes in children with asthma. Psychosom Med.

[28] Ebrecht M, Hextall J, Kirtley L, Taylor A, Dyson M, Weinman J (2004). Perceived stress and cortisol levels predict speed of wound healing in healthy male adults. Psychoneuroendocrinology.

[29] Sabbah W, Watt RG, Sheiham A, Tsakos G (2008). Effects of allostatic load on the social gradient in ischaemic heart disease and periodontal disease: evidence from the Third National Health and Nutrition Examination Survey. J Epidemiol Community Health.

[30] Seeman TE, McEwen BS, Rowe JW, Singer BH (2001). Allostatic load as a marker of cumulative biological risk: MacArthur studies of successful aging. Proc Natl Acad Sci U S A.

[31] Juster R, Hatzenbuehler ML, Mendrek A, Pfaus JG, Smith NG, Johnson PJ, Lefebvre-Louis J, Raymond C, Marin M, Sindi S, Lupien SJ, Pruessner JC (2015). Sexual orientation modulates endocrine stress reactivity. Biol Psychiatry.

[32] Hatzenbuehler ML, McLaughlin KA (2014). Structural stigma and hypothalamic-pituitary-adrenocortical axis reactivity in lesbian, gay, and bisexual young adults. Ann Behav Med.

[33] DuBois LZ, Gibb JK, Juster R, Powers SI (2021). Biocultural approaches to transgender and gender diverse experience and health: integrating biomarkers and advancing gender/sex research. Am J Hum Biol.

[34] Colizzi M, Costa R, Pace V, Todarello O (2013). Hormonal treatment reduces psychobiological distress in gender identity disorder, independently of the attachment style. J Sex Med.

[35] DuBois LZ, Powers S, Everett BG, Juster R (2017). Stigma and diurnal cortisol among transitioning transgender men. Psychoneuroendocrinology.

[36] Tan KK, Treharne GJ, Ellis SJ, Schmidt JM, Veale JF (2020). Gender minority stress: a critical review. J Homosex.

[37] Olson KR, Durwood L, DeMeules M, McLaughlin KA (2016). Mental health of transgender children who are supported in their identities. Pediatrics.

[38] Hill DB, Menvielle E, Sica KM, Johnson A (2010). An affirmative intervention for families with gender variant children: parental ratings of child mental health and gender. J Sex Marital Ther.

[39] Cook SH, Pruessner JC, Lupien SJ, Juster R (2018). Sexual orientation moderates the association between parental overprotection and stress biomarker profiles. Psychol Sex.

[40] Higa D, Hoppe MJ, Lindhorst T, Mincer S, Beadnell B, Morrison DM, Wells EA, Todd A, Mountz S (2012). Negative and positive factors associated with the well-being of lesbian, gay, bisexual, transgender, queer, and questioning (LGBTQ) youth. Youth Soc.

[41] Bhattacharya N, Budge SL, Pantalone DW, Katz-Wise SL (2020). Conceptualizing relationships among transgender and gender diverse youth and their caregivers. J Fam Psychol.

[42] (2018). AAP Policy Statement Urges Support and Care of Transgender and Gender-Diverse Children and Adolescents. HealthyChildren.

[43] Jessop DS, Turner-Cobb JM (2008). Measurement and meaning of salivary cortisol: a focus on health and disease in children. Stress.

[44] Wolf JM, Nicholls E, Chen E (2008). Chronic stress, salivary cortisol, and alpha-amylase in children with asthma and healthy children. Biol Psychol.

[45] Calcaterra V, Vinci F, Casari G, Pelizzo G, de Silvestri A, De Amici M, Albertini R, Regalbuto C, Montalbano C, Larizza D, Cena H (2019). Evaluation of allostatic load as a marker of chronic stress in children and the importance of excess weight. Front Pediatr.

[46] Grant JM, Motter LA, Tanis J (2011). Injustice at every turn: a report of the national transgender discrimination survey. National Center for Transgender Equality.

[47] Villanti AC, Johnson AL, Ilakkuvan V, Jacobs MA, Graham AL, Rath JM (2017). Social media use and access to digital technology in US young adults in 2016. J Med Internet Res.

[48] Dimant OE, Cook TE, Greene RE, Radix AE (2019). Experiences of transgender and gender nonbinary medical students and physicians. Transgend Health.

[49] Ozer E (2016). Youth-led participatory action research: developmental and equity perspectives. Adv Child Dev Behav.

[50] Hidalgo MA, Chen D, Garofalo R, Forbes C (2017). Perceived parental attitudes of gender expansiveness: development and preliminary factor structure of a self-report youth questionnaire. Transgend Health.

[51] Felitti VJ, Anda RF, Nordenberg D, Williamson DF, Spitz AM, Edwards V, Koss MP, Marks JS (1998). Relationship of childhood abuse and household dysfunction to many of the leading causes of death in adults. Am J Prev Med.

[52] Côté-Lussier C, Jackson J, Kestens Y, Henderson M, Barnett TA (2015). A child's view: social and physical environmental features differentially predict parent and child perceived neighborhood safety. J Urban Health.

[53] Côté-Lussier C, Mathieu M, Barnett TA (2015). Independent associations between child and parent perceived neighborhood safety, child screen time, physical activity and BMI: a structural equation modeling approach. Int J Obes (Lond).

[54] (2012). Mental health screening and assessment tools for primary care. American Academy of Pediatrics.

[55] Testa RJ, Habarth J, Peta J, Balsam K, Bockting W (2015). Development of the gender minority stress and resilience measure. Psychol Sex Orient Gender Diver.

[56] Gordis EB, Granger DA, Susman EJ, Trickett PK (2006). Asymmetry between salivary cortisol and alpha-amylase reactivity to stress: relation to aggressive behavior in adolescents. Psychoneuroendocrinology.

[57] Hankin BL, Badanes LS, Smolen A, Young JF (2015). Cortisol reactivity to stress among youth: stability over time and genetic variants for stress sensitivity. J Abnorm Psychol.

[58] Kong SY, Stabler TV, Criscione LG, Elliott AL, Jordan JM, Kraus VB (2006). Diurnal variation of serum and urine biomarkers in patients with radiographic knee osteoarthritis. Arthritis Rheum.

[59] Birch L, Anzman-Frasca S, Paul IM (2012). Starting early: obesity prevention during infancy. Nestle Nutr Inst Workshop Ser.

[60] Lippold MA, Davis KD, McHale SM, Buxton OM, Almeida DM (2016). Daily stressor reactivity during adolescence: the buffering role of parental warmth. Health Psychol.

[61] Noppe G, Van Rossum EF, Koper JW, Manenschijn L, Bruining GJ, de Rijke YB, van den Akker EL (2014). Validation and reference ranges of hair cortisol measurement in healthy children. Horm Res Paediatr.

[62] Vanaelst B, Michels N, De Vriendt T, Huybrechts I, Vyncke K, Sioen I, Bammann K, Rivet N, Raul J, Molnar D, De Henauw S (2013). Cortisone in hair of elementary school girls and its relationship with childhood stress. Eur J Pediatr.

[63] Ice GH (2007). Measuring emotional and behavioral response. Measur Stress Hum.

[64] McEwen BS, Milner TA (2017). Understanding the broad influence of sex hormones and sex differences in the brain. J Neurosci Res.

[65] Abraham GE, Maroulis GB (1975). Effect of exogenous estrogen on serum pregnenolone, cortisol, and androgens in postmenopausal women. Obstet Gynecol.

[66] Hermans EJ, Ramsey NF, van Honk J (2008). Exogenous testosterone enhances responsiveness to social threat in the neural circuitry of social aggression in humans. Biol Psychiatry.

